# Inferences About Radionuclide Mobility in Soils Based on the Solid/Liquid Partition Coefficients and Soil Properties

**DOI:** 10.1007/s13280-013-0408-4

**Published:** 2013-04-26

**Authors:** Gustav Sohlenius, Peter Saetre, Sara Nordén, Sara Grolander, Steve Sheppard

**Affiliations:** 1 Geological Survey of Sweden, Box 670, 751 28 Uppsala, Sweden; 2 Swedish Nuclear Fuel and Waste Management Co. (SKB), Box 250, 101 24 Stockholm, Sweden; 3 Facilia AB, Gustavslundsvägen 151C, 167 51 Bromma, Sweden; 4 ECOMatters Inc., Box 430, Pinawa, MB R0E 1L0 Canada

**Keywords:** Solid/liquid partition coefficient, Radionuclides, Soil chemistry, Arable land, Regolith

## Abstract

To assist transport modeling in assessments of the radiological impact of a geological repository for radioactive wastes, the mobility of various elements was studied in arable and wetland soils in the Forsmark region, Sweden. Pore water and total element contents were determined for five types of unconsolidated deposits (regolith), spanning a wide range of soil properties with respect to pH and organic matter content. Two soil depths were sampled to capture element mobility in regolith layers affected and unaffected by soil-forming processes. The solid/liquid partition coefficients (*K*_d_ values) for most elements varied significantly among regolith types. For most elements, the observed variations in *K*_d_ values could be explained by variations in soil properties. For many elements, mobility increased with decreasing soil pH. The results provide a significant addition of data on radionuclide retention in soils, taking account of soil properties and processes.

**Electronic supplementary material:**

The online version of this article (doi:10.1007/s13280-013-0408-4) contains supplementary material, which is available to authorized users.

## Introduction

This paper addresses whether the current chemical properties of arable land can be used to predict the properties of future cultivated areas, and how such predictions can be used in safety assessments. Specifically, it examines the chemical properties of different types of cultivated regolith (unconsolidated deposit), with a focus on the solid/liquid partition coefficients (*K*_d_ values) that are used to evaluate the mobility of elements in different soil types. The *K*_d_ value is the ratio of the concentration of an element on a solid phase (soil or sediment, C_s_, mg kg^−1^ dw, or Bq kg^−1^ dw) divided by the equilibrium concentration in the contacting liquid phase (water, C_w_, mg m^−3^, or Bq m^−3^). Thus, a large *K*_d_ value implies that most of an element is adsorbed to solids and consequently that its mobility due to water flow is low. *K*_d_ values are commonly used in transport models as a mean to represent retention of contaminant radionuclides in soils (Gil-García et al. 2009; Vandenhove et al. 2009). The *K*_d_ values are traditionally empirically measured for a specific site, and represent the long-term equilibrium concentrations that result from sorption or attenuation on soil or sediment solids. *K*_d_ values in this context encompass more than simple exchange or sorption reactions, they may also reflect chemical precipitation and biological incorporation. In other words, *K*_d_ values represent the partitioning between soil solution and soil solids, and can be used to estimate elemental mobility in different types of regolith. *K*_d_ values are dependent on a number of soil properties such as pH, and the content of clay and organic matter (Sheppard et al. 2011). Thus, by developing robust relationships between *K*_d_ values and soil properties, one can predict the expected range of *K*_d_ values for specific soil conditions, and determine how the *K*_d_ values of existing soils are likely to change in the future.

The Swedish Nuclear Fuel and Waste Management Company (SKB) has made a safety assessment for a geological repository for radioactive waste, planned to be situated close to Forsmark at the coast of the Baltic Sea (Kautsky et al. 2013). In this safety assessment the exposure to humans due to intake of contaminated crop is an important contribution to the total exposure (Saetre et al. 2013). Furthermore, arable land is often situated in discharge areas for groundwater, i.e., former wetlands, where water from the deep bedrock may reach the surface system. In the case of a repository for long-lived radionuclides, the relevant time period for radiological impact assessment is several tens of thousands of years or longer. An issue arising from this is how the chemical properties of arable land will change during this long period of time. Element mobility will affect accumulation in soil and subsequent uptake of radionuclides in crops consumed by humans. The available areas used for cultivation will increase as a consequence of new land rising from the sea due to the land upheaval. Organic soils on land used for agriculture will evolve to mineral soils as a consequence of oxidation. The regolith in the Forsmark area that potentially can be used as arable land was deposited in a similar way and has similar properties to most other deposits presently used as arable land in Sweden. By studying properties (e.g., *K*_d_ values) of the Forsmark soils it is consequently possible to draw conclusions regarding soils, which are used for cultivation in other parts of Sweden.

In this paper, we discuss how elemental mobility (as characterized by *K*_d_ values) varies with soil properties in five regolith types investigated by Sheppard et al. (2011). The *K*_d_ values are correlated with soil properties by the use of regression models. The results are further used to discuss how the soil properties, and thereby element mobility, may change during the forthcoming several tens of thousands of years.

## Materials and Methods

### The Studied Site

At present, Forsmark is situated on the coast of the Baltic Sea (Fig. S1 in Electronic Supplementary Material). The area is dominated by forested glacial till, and is only partly cultivated. However, in a few thousand years, large areas of clays, suitable for cultivation, will be uplifted. Since the future arable land is situated, to a large extent, at the present sea floor, the chemical properties of equivalent deposits presently used as arable land were used in this study. In the safety assessment the evolution of agricultural land in a far future was studied through modeling the shoreline displacement and landscape development of the area (Brydsten and Strömgren 2010; Lindborg et al. 2013).

### Soil Sampling and Analyses: Choice of Sampling Locations

To identify sites that would be representative of arable land in the future Forsmark area, potential areas were examined in the GIS software ArcGIS 9.3. First the distribution of regolith in areas presently used as arable land in the county of Uppsala was determined. For that we used the SGU maps of regolith. The results were compared with the modeled future distribution of regolith (Brydsten and Strömgren 2010) in order to identify the regolith types that represent the arable land of the future. The focus was on areas with potential for discharge of deep groundwater. Five regolith types, all situated within 20 km of the Forsmark area, were identified and sampled; clayey till, glacial clay, clay gyttja and two peat types. The sampled sites are all situated in areas that were uplifted within the last few thousand years. The peat samples were both from cultivated areas (hereafter named cultivated peat samples) and from undisturbed wetlands (hereafter named wetland peat samples) that may be cultivated in the future. Five sites were sampled from each regolith type. Samples were taken at two different depths at each position, one at 20–25 cm below the ground surface and one 50–55 cm below the ground surface. At the sites used as arable land, the uppermost sample was taken within the zone affected by plowing, whereas the lowermost sample was taken at a depth that was assumed to be more or less unaffected by soil-forming processes (i.e., the soil parent material). At the sites used as arable land, samples were taken in spade dug pits whereas a peat corer was used in the wetlands (Fig. S2 in Electronic Supplementary Material). At each site, samples were taken from five sampling positions. The five samples from each sampled level were mixed to form one composite sample that was used for further analyses.

### *K*_d_ Values and Soil Chemical Properties

To calculate *K*_d_ values, concentrations of elements in both the pore water and solid phase were measured. For measurements of the pore water, the samples were saturated with water to represent field capacity and thereafter incubated for 1 week at room temperature. A week was considered appropriate because it is consistent with the time scale of natural in-field wetting and drying cycles. For the solid phase, samples were analyzed following digestion with aqua regia. The use of aqua regia is a compromise intended to extract the elements in soil that may be subject to release by weathering in an environmentally relevant time scale of decades or centuries. The extraction includes exchangeable and acid soluble phases but not phases locked in silicate minerals. A separate preparation method was applied to determine Cl, Br, I, and ^226^Ra content. All elemental concentrations were determined using inductively coupled plasma sector field mass spectrometry (ICP-SFMS).

All measured soil properties are listed in Table 1. In a SKB-report by Sheppard et al. (2011) the methodology and data presented here are thoroughly described. The statistical analysis and interpretations presented in this paper were not included in the report by Sheppard et al. (2011).

**Table 1:** Measured soil properties with a brief reference to the methods used

Soil parameter	Method
pH	pH was measured in H_2_O, CaCl_2_, and KCl
Acidity	1. Extraction in NH_4_-acetate and titration with NaOH
Exchangeable base cations	2. Analyzed with ICP-AEA after extraction in NH_4_-acetate
Cation exchange capacity (CEC)	Sum of 1 and 2
Grain size composition	Particles >0.2 mm sieving Particles <0.2 mm sedimentation
Organic carbon content	Analyzed after combustion (LECO)
Inorganic carbon content	Gas pressure after treatment with HCl
Total N	Analyzed after combustion (LECO)
Content of organic material	Loss on ignition at 550 °C
Element concentration in solid phase	Analyzed with ICP-SFMS after extraction with aqua regia (most elements)
Element concentration in pore water	Analyzed with ICP-SFMS after incubation

### Statistical Analysis

To explore the correlation structure of soil characteristics (other than *K*_d_), and to display how patterns in soil characteristics varied with soil type and depth, we analyzed measured characteristics with a principal component analysis (PROC PRIN). Principal components (PCs) were derived from standardized variables (unit variance), and missing values were imputed as being half of the detection limit. Graphs based on loadings and scores associated with the first two components were used to summarize the patterns.

The effects of soil type and soil depth on *K*_d_ values were tested using a mixed linear model for a split plot experiment (PROC MIXED). In the model, soil type and depth, and the interaction between soil type and depth, were treated as fixed factors whereas sampling location within a soil type was treated as a random factor. Thus, for the test of soil types the sampling site was used as the level of replication (*n* = 25), whereas for the tests of an effect of soil depth, and the dependence of the depth effect on soil type, the soil samples were used as the level of replication (*n* = 50).

To examine to what degree the observed variation in *K*_d_ values could be explained by variations in soil characteristics, we used multiple linear regression. To avoid instability in estimates of *β*-coefficients, caused by co-linearity in the explanatory variables, sets of strongly correlated variables (*r* > 0.7) were represented by one of the explanatory variables only. Thus the *K*_d_ value for each element was modeled with forward stepwise linear regression using pH (water), organic C, CEC, acidity, exchangeable K, Na, and aluminum, and clay content as explanatory variables (PROC REG). No imputation of missing values was performed for the split-plot and the regression analyses.

To facilitate the graphical presentation of multi-element results, elements were divided into disjoint clusters with respect to similarity in *K*_d_ values across the 50 samples. We used the VARCLUS algorithm, which maximizes the variance explained by cluster components (summed over all formed clusters), and used the first PCs (based on the correlation matrix) of each cluster as cluster components (PROC VARCLUS). *K*_d_ values of the lanthanides were strongly correlated, and thus we grouped them into a cluster of their own before the analysis (i.e., these elements were not included in the cluster analysis).

As *K*_d_ values are the ratio of two measurements the distribution is inherently positively skewed. To achieve approximately normally distributed residuals, *K*_d_ values were transformed to a logarithmic scale prior to analysis. All statistical analyses were performed with the SAS/STAT^®^ software, version 9.3 (SAS Institute Inc., Cary, NC, USA).

## Results

**Fig. 1:**
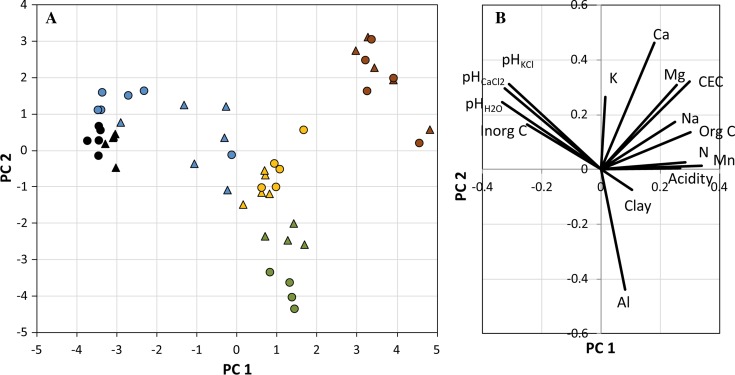
Patterns in soil chemical characteristics in agricultural soils and natural wetlands in the Forsmark region. Calcium, K, Mg, Na, and Al refer to results from analyses of exchangeable elements (Table 1). The first two principal components (PCs) explained 45 and 21 % of the total variation in soil characteristics, respectively. **a** Scores of regolith samples along the first two PCs. *Symbol colors* indicate regolith type (*black* clayey till, *blue* glacial clay, *green* clay gyttja, *yellow* cultivated peat, *brown* wetland peat) and shape sampling depth (*triangle* 20 cm, *circle* 50 cm). **b** PC loadings of chemical properties along the first two principal components

Many soil properties varied significantly among the examined regolith types (Table 2). As soil characteristics tend to vary in concert, 66 % of the variation in soil properties could be explained by only two underlying PCs. These components were primarily related to regolith type. That is, clay soils separated from the wetland peat along the first PC (Fig. 1a), which reflected a gradient of decreasing pH and inorganic C content and increasing concentrations of organic carbon, acidity, and exchangeable cations. Clay gyttja separated from the other regolith types along the second PC, which reflected a gradient of decreasing aluminum content, and increasing pH and levels of exchangeable cations (K, Mg, and Ca) unrelated to organic matter content. Interestingly, this gradient was also related to soil depth, but the relationship clearly depended on regolith type. That is, the deep clay gyttja samples had consistently lower values along PC2 than the corresponding upper samples from cultivated soil, whereas the pattern was the opposite in glacial clay, and no separation was apparent among the other soils.

**Table 2:** Selected soil properties of the studied regolith types (modified from Sheppard et al. 2011). Mean values ± standard deviations are listed. Values from more than 2000 samples from the uppermost 20 cm of Swedish arable soils have been added as a reference (Eriksson et al. 2010)

Regolith type	Dry bulk density (g cm^−3^)	Organic content (%)	pH in H_2_O	Clay <2 μm (% of mineral fraction)	Total S (%)
Clay till	1.8 ± 0.2	3.4 ± 1.5	8.1 ± 0.29	17 ± 5.0	0.036 ± 0.029
Clay gyttja	0.67 ± 0.13	16 ± 4.4	4.8 ± 0.45	46 ± 15	0.27 ± 0.073
Glacial clay	1.3 ± 0.35	6.5 ± 5.9	7.0 ± 0.61^a^	35 ± 9.9^a^	0.10 ± 0.081
			8.0 ± 0.67	56 ± 9.2	
Cultivated peat	0.24 ± 0.06	81 ± 6.5	6.0 ± 0.16	–	0.56 ± 0.14
Wetland peat	–	91 ± 3.6	5.7 ± 0.78	–	0.75 ± 0.27
Average Swedish arable land^b^	–	4.1 ± 6.6	6.3 ± 0.6	–	0.06 ± 0.11

^a^Data for 20 cm (above) and 50 cm (below) depth are given separately for pH and clay content in the glacial clay because these properties vary significantly with depth

^b^Data from Eriksson et al. (2010)

**Fig. 2:**
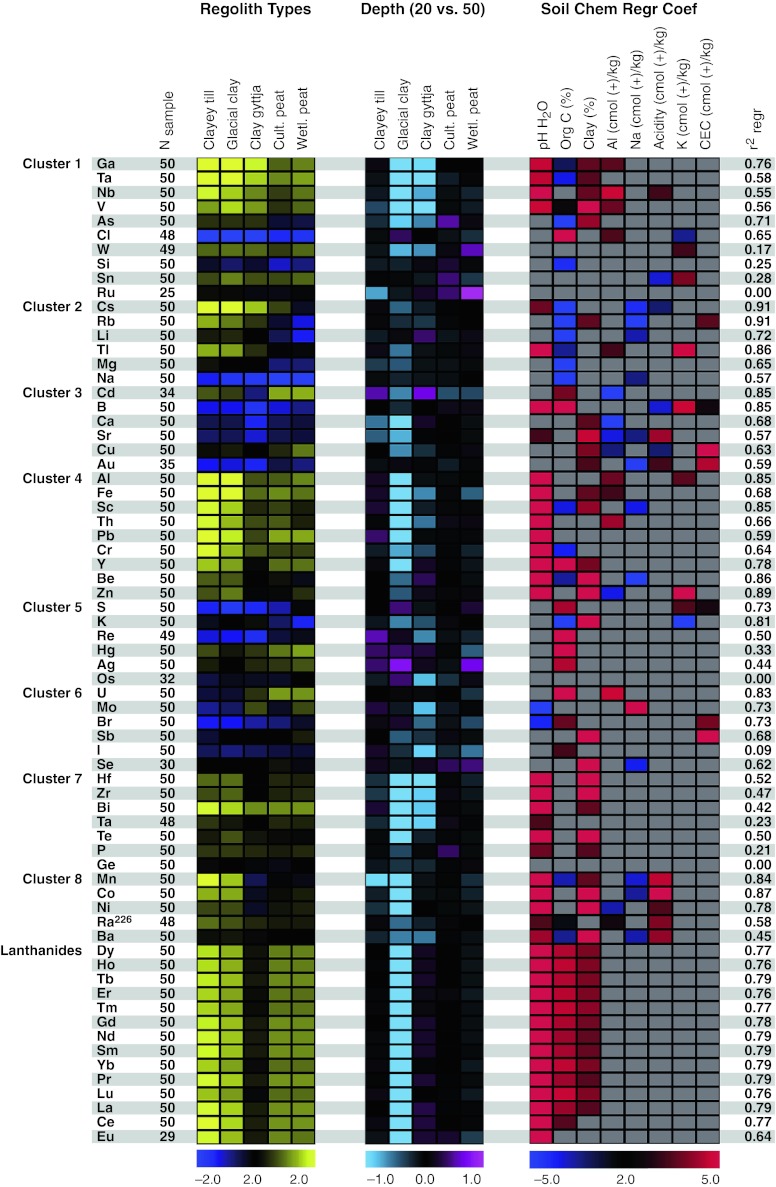
Relationship between *K*_d_ values, regolith type, sampling depth, and soil properties for 69 elements in agricultural soils and natural wetland peat in the Forsmark region. Elements have been grouped into clusters with respect to similarity in *K*_d_ profile. *Left panel* *K*_d_ values as a function of regolith type over both sampled depths. *Yellow* represents high *K*_d_ whereas *blue* represents low *K*_d_ values. *Central panel* Difference in *K*_d_ between the upper and lower sample within each regolith type. *Purple* represents a higher *K*_d_ in the top layer, whereas *light blue* indicates a higher *K*_d_ in the deep soil layer. For both the *left* and *central panel*, *K*_d_ is expressed on a logarithmic scale where a unit change represents a tenfold difference. *Right panel* Effect of soil characteristics on *K*_d_ values. *Color* represents the predicted strength of soil characteristics (i.e., *t* values for *β*-coefficients) given the full regression model. *Red* represents a positive relationship, *dark blue* a negative. For many elements there is a positive relationship between soil pH and *K*_d_. The results shown in this figure are presented in detail in Appendix 1 (Electronic Supplementary Material)

Using a threshold of *p* < 0.01 as an indicator of a significant effect (corresponding to less than one expected false positive from 69 tests), 56 of the 69 examined *K*_d_ values (81 %) were affected by regolith type. Though the effect of regolith types on *K*_d_ values varied substantially among individual elements, co-variation among elements was clearly captured in the identified clusters, and some general trends could be seen in the data (Fig. 2; Appendix 1 in Electronic Supplementary Material). That is, more than half of the elements showed a systematic mean difference between regolith types that were rich (peats) or poor (glacial clay, clayey till, and clay gyttja) in organic matter, and peat soils tended to have lower *K*_d_ values. Whereas *K*_d_ values tended to be similar in cultivated and natural peat soils (only one out of seven elements differed), *K*_d_ values for more than half of the elements varied substantially among the three clay soils. Not surprisingly, it was the *K*_d_ value in the clay gyttja that most frequently stood out from the other two regolith types, and clay gyttja tended to have lower *K*_d_ values than the other two clay soils (Fig. 2; Appendix 1 in Electronic Supplementary Material).

There were no clear depth effects that were consistent across all five regolith types. Instead, the magnitude and direction of *K*_d_ changes with depth varied with regolith type. *K*_d_ of 50 of the elements (72 %) showed a clear depth effect in glacial clay, and *K*_d_ was consistently lower in the cultivated layer as compared with the underlying parent material. There was a similar but less pronounced trend in clay gyttja, and 14 elements differed between the cultivated layer and the parent material. However, for clayey till, cultivated peat, and wetland peat the effect of depth was limited or non-existent (four, three, and zero elements differed at the *p* < 0.01 level) and there was no tendency towards either increasing or decreasing *K*_d_ values with increasing soil depth.

Measured soil properties could explain a large fraction of the variation in *K*_d_ values among soil samples (Fig. 1b). That is, for 55 elements soil characteristics explained more than half of the observed variation in *K*_d_ values, and for 29 elements more than three quarters of *K*_d_ variation was explained by the regression model. The three soil characteristics with most explanatory power were pH, organic C content, and clay content (Fig. 2; Appendix 1 in Electronic Supplementary Material). That is, pH affected the *K*_d_ values of 44 elements (64 %) and the average absolute *t* value for the associated beta coefficients (i.e. |*β*|/SE) was 8.3, whereas the corresponding values for organic carbon and fine fraction clay content were 42 and 5.0, and 39 and 3.8, respectively. For a majority of elements, *K*_d_ values increased with pH and clay content. Certain elements showed increased *K*_d_ value with increased organic matter content, whereas other elements showed the opposite (Fig. 2).

## Discussion

The regolith types were selected to represent wetlands and a range of cultivated soils that may be contaminated by radionuclides. Before we discuss how element mobility (*K*_d_) varies as an effect of soil properties, we briefly describe the five sampled regolith types (see also Box 1; Table 2).

**Box 1:** Soil properties and element mobility characteristics of agricultural soils and natural wetlands in the Forsmark region

Parameter	Wetland peat	Cultivated peat	Clay gyttja	Glacial clay	Clayey till
Groundwater	Fens with the ground water table near the ground surface	Former fens with an artificially lowered groundwater table	Former fens with an artificially lowered groundwater table	Flat to gently sloping areas which in some areas may be former fens with artificially lowered groundwater table	Gently undulating to flat areas with a deep laying groundwater surface
Redox conditions	Reducing conditions	Oxidizing conditions in uppermost layer	Oxidizing conditions in uppermost layer	Oxidizing conditions	Oxidizing conditions
Organic content	High organic content	High organic content (lower than in wetland peat)	Relatively low organic carbon content	Low organic carbon content, contains inorganic carbon (from CaCO_3_)	Low organic carbon content, contains inorganic carbon (from CaCO_3_)
pH	Medium pH	Medium pH	Low pH	High pH	High pH
Density	Low density	Low density (higher than wetland peat)	Medium density	High density (lower than clayey till)	High density
*K*_d_ value	High *K*_d_ values for many elements	High *K*_d_ values for many elements	Low *K*_d_ values for most elements	High *K*_d_ values for most elements	High *K*_d_ values for most elements
*K*_d_ variations within the soil			For some elements significant differences in *K*_d_ values with depth	For many elements significant differences in *K*_d_ values with depth	

### Wetland Peat

**Fig. 3:**
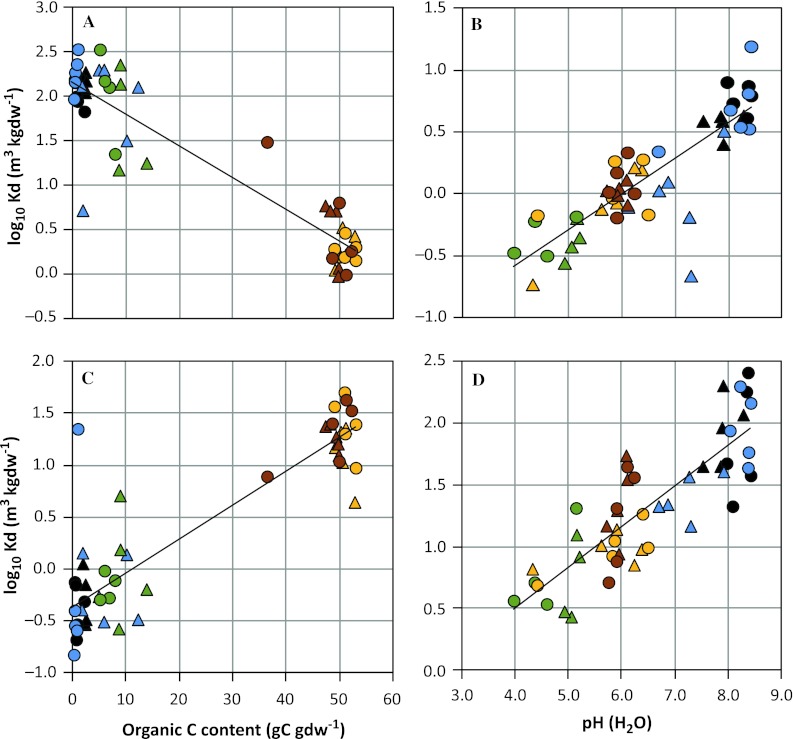
*K*_d_ values for four selected elements as a function of soil chemical characteristics in agricultural soils and natural wetlands in the Forsmark region, **a** Cesium, **b** Nickel, **c** Uranium, **d** ^226^Radium. The *line* represents the modeled relationship between *K*_d_ and soil C content or pH, whereas *symbols* represent observed *K*_d_ values corrected for systematic variation explained by other soil characteristics included in the regression model (see Fig. 2, right panel). The regolith types are symbolized by *different colors*, whereas sampling depth is indicated by *symbol shape* (see legend in Fig. 2)

Peat is formed from incomplete decomposition of plant materials in anoxic wetland conditions. The studied wetland sites were uplifted above sea level 1500 to 2000 years ago and since then a ca. 1 m thick peat layer has formed. The groundwater table is situated close to the ground surface and the water content is high, and reducing conditions may consequently occur close to the ground surface. Most of the studied soil properties and *K*_d_ values for almost all elements did not vary systematically between surface and deep samples (Figs. 1a, 3).

### Cultivated Peat

These sampled sites were situated in former wetlands where the groundwater table has been artificially lowered. This has caused oxidizing conditions and subsidence of the uppermost peat layers (cf. Berglund 1996). The organic content in the cultivated peat is somewhat lower than in the wetland peat, reflecting enrichment of minerogenic material as a consequence of oxidation of organic material. The sites have been uplifted for a longer period than the other sites (up to 5000 years), which reflects that peat can accumulate for centuries before used for cultivation. Most of the studied soil properties and *K*_d_ values for almost all elements did not vary systematically between the cultivated layer and the deep samples (Figs. 1a, 3). Moreover, for most elements there were no large differences in *K*_d_ values between the cultivated and wetland peat, implying that the conversion of wetland to arable land does not affect elemental mobility significantly.

### Clay Gyttja

Clay gyttja occurs in the lowest topographical parts of the landscape (Persson 1985) and cultivated clay gyttja is often situated in former fens. Studies of clay gyttja have shown that these deposits often contain iron sulfides below the groundwater table (Åström 1998; Sohlenius and Öborn 2004). When clay gyttja areas are cultivated, the groundwater table is lowered and, if sulfides are present, oxidation will cause a decrease in pH. As the sampled soils were moderately rich in total sulfur, sulfide oxidation is likely to explain the observed low pH. Moreover, as the soil chemical profile of the cultivated layer showed a small but consistent difference from deeper samples (Fig. 1), it was not surprising that the mobility of almost a fifth of the elements differed significantly between layers.

### Glacial Clay

Glacial clay (Fig. S2 in Electronic Supplementary Material) is often situated in higher topographical areas than peat and clay gyttja. The glacial clay is low in organic carbon but contains inorganic carbon, which comes from the carbonate parent materials (Persson 1985) and explains the high pH in the samples. Several of the measured soil properties show significant differences between the upper and lower samples. The inorganic carbon content and pH are lower in the uppermost samples, which indicate that CaCO_3_ has leached out. Furthermore, the clay content is lower and sand content higher in the uppermost samples.

### Clayey Till

Compared with the other deposits, clayey till is generally situated in dryer and higher topographical areas (Fig. S2 in Electronic Supplementary Material). Many properties of clayey till are similar to these of glacial clay (Box S1 in Electronic Supplementary Material), including a relatively high pH caused by the presence of CaCO_3_ minerals. The clayey till shows similar *K*_d_ values to those of the glacial clay, with relatively high values for most elements. The clayey till soils are young and have not been subjected to chemical weathering long enough for the CaCO_3_ to be fully leached. However, the lower content of inorganic carbon in the uppermost samples is probably an effect of calcite weathering.

### Soil Properties and *K*_d_

Clearly, the strongest determinant of element mobility in the studied soils was pH (Figs. 1, 2). That is, variations in *K*_d_ values between and within regolith types could to a large extent be explained by co-variation in pH. The lowest *K*_d_ values were generally found in the samples with the lowest pH, which means that the mobility for most elements increases with decreasing pH. This was expected, as low pH is associated with increase rates of chemical weathering and earlier studies have shown that many trace elements are mobilized from the relatively acid soils that often develop in cultivated areas with clay gyttja (Sohlenius and Öborn 2004). One reason for the relatively strong relationship between pH and *K*_d_ is the wide span of pH values in the studied regolith types.

The amount of organic carbon was also an important determinant of element mobility, and variations in organic matter content had a significant influence on *K*_d_ values for about half of the analyzed elements. This was expected as mineral (clayey till, glacial clay, and clay gyttja) and peat soils have few common properties. Besides their pronounced differences in organic matter content, the examined peat soils differed from the mineral soils also with respect to N content, clay content, cation exchange capacity, and the concentration of extractable base cations (e.g., K, Ca, Mg). These differences tended to be more pronounced in the samples from the natural wetlands.

Contrary to the case with pH, the effects of carbon content on element mobility were both positive and negative. For Cd and U (Fig. 3b) the *K*_d_ values in the peat were more than tenfold higher than in the basic mineral soils, whereas Cs (Fig. 3a) showed the opposite trend. Cs and Rb (Fig. 2), and to a lesser extent the related element K, behaved distinctly differently between minerogenic and peat soils. These elements are noted for being retained within layers of clay minerals, resulting in especially high *K*_d_ values in clay soils. Elements such as Cl, I, and S are expected to have higher *K*_d_ values in organic soils than minerogenic soils because of incorporation into organic material created by photosynthesis in plants. The *K*_d_ values for S in the peat studied here were higher than in the mineral soils. However, no such trend was found for Cl and I, and the *K*_d_ values for these elements were not correlated with any of the measured soil properties.

When comparing *K*_d_ values between the studied soils, it should be remembered that the peat soils have dry bulk densities that are about sixfold lower than the mineral soils (Table 1). Thus, in terms of the ability of a given soil volume to retain an element with a specific *K*_d_ value, peat is less retentive than mineral soils.

The soils studied here embrace most soils properties of Swedish soils used for agriculture (Eriksson et al. 2010). Thus, the results presented here have implications for the bioavailability of potentially harmful trace elements occurring naturally in Swedish arable soils. Results from studies of some trace elements have shown that there is a negative correlation between the contents of, e.g., Cd and Ni in plants and soil pH (Palko 1986; Eriksson 1990). The low *K*_d_ values for these elements in the acid clay gyttja soils studied here indicate a potential for high uptakes by plants.

### Future Development of the Studied Soils

The studied soils were relatively young and unaffected by weathering processes; further changes of soil properties, which will affect *K*_d_, are likely to occur during the forthcoming thousands of years.

The clay gyttja studied here have probably not been cultivated for long and are, therefore, still characterized by low pH and high mobility for many elements (low *K*_d_ values), due to oxidation of sulfide minerals. However, *K*_d_ and pH will increase as the sulfides oxidize and acid is leached out from the soils. Studies from Finland by Österholm and Åström (2004) showed that the leaching of elements from oxidized sulfidic sediment is reduced by 50 % after 30 years of cultivation, due to decreasing acidity from sulfide oxidation.

Glacial clay and clayey till are rich in calcite and are consequently characterized by both high pH and high *K*_d_ values. As long as calcite is present, the soil pH is buffered, but as the last calcite is dissolved the buffering disappears and pH can than decrease rapidly. The calcite-rich soils studied here have been uplifted for 2000 years or less and show lower contents of inorganic carbon in the uppermost samples indicating that the calcite has been leached out to a significant extent. That process will continue and, in a few thousand years, will cause decreasing pH and *K*_d_ values. An earlier study from the same region as Forsmark (Ingmar and Moreborg 1976) has shown that calcite leaches completely from the uppermost part of till that has been exposed to weathering for a few thousand years.

Cultivated peat is characterized by relatively high *K*_d_ values for many elements, but these elements may be mobilized due to oxidation of the peat. There are several studies showing that cultivated peat oxidizes rapidly and that the thickness may decrease by more than 10 mm/year (Kasimir-Klemedtsson et al. 1997). These processes may cause mobilization of elements bound in the peat and will also expose underlying deposits, often sulfidic clay gyttja, meaning that the *K*_d_ properties of the soil will change drastically as the peat oxidizes.

### Implication for the SKB Safety Assessment

For SKB’s safety assessment, the succession of landscape and future land uses during the forthcoming thousands of years are of major importance for the radionuclide modeling (Avila et al. 2013; Piqué et al. 2013). The regolith types studied here are most probably equivalent to the deposits that in the future might be cultivated and possibly affected by radionuclides (Avila et al. 2013). Cultivation of organic soil where radionuclides have accumulated for a long period of time is likely to be a major exposure pathway for human inhabitants (Saetre et al. 2013). Thus, it is important that a safety assessment have a good representation of transport and accumulation in soils with a potential for cultivation located in areas where contaminated groundwater may be discharged. The studied soils give rise to a large range of *K*_d_ values and it is not reasonable to apply the full range to model radionuclide transport in all agricultural soils (Avila et al. 2013). Instead, the *K*_d_ values should be matched with the modeled geographical distribution of different regolith types and land uses (Lindborg et al. 2013) in relation to the probable discharge areas for deep groundwater (Berglund et al. 2013). Peat and clay gyttja commonly occur in discharge areas and the properties of arable land on these regolith types are consequently of large importance for the safety assessment. Glacial clay and clayey till are more often situated in recharge areas and the properties of these deposits are of more relevance in scenarios in which contaminated irrigation water is the focus of attention.

## Conclusions

The *K*_d_ values applicable to the present arable land can be expected to be appropriate also to future arable land. However, the properties of most regolith types will change through time due to weathering and oxidation of organic material. The studied regolith types were characterized by distinctly different properties, and these explain the range of *K*_d_ values obtained for the various elements studied.

Some elements that are relevant for SKB’s safety assessment show relatively low *K*_d_ values (implying high mobility) in peat and clay gyttja. These deposits commonly occur in discharge areas for groundwater and *K*_d_ values of peat and clay gyttja are therefore of importance for the safety assessment.

The single most important factor controlling *K*_d_ values is pH and many elements had low *K*_d_ values in the clay gyttja soils characterized by a low pH. It is consequently possible to predict the future mobility of different elements by predicting the future changes in pH in different regolith types.

Another important factor explaining *K*_d_ values is the organic content of the soils, and there are both negative and positive correlations between organic carbon and *K*_d_. Peat is almost completely built up of organic matter and peat can only be cultivated for a relatively short period of time due to oxidation and compaction. That oxidation may cause mobilization of radionuclides and other elements bound in the peat.

The results presented here give information regarding the mobility of potentially harmful elements that are present in agricultural soils of today. That gives in turn an indication of the potential for high uptake by crops growing on soils with different properties, which is relevant for the SKB safety assessments.

## Electronic supplementary material

Below is the link to the electronic supplementary material.

Supplementary material 1 (PDF 1105 kb)
