# Supplementary material for: Inferences About Radionuclide Mobility in Soils Based on the Solid/Liquid Partition Coefficients and Soil Properties
Source: Ambio. 2013 Apr 26;42(4):414–24. doi: 10.1007/s13280-013-0408-4 (PMC3636366; doi:10.1007/s13280-013-0408-4)
Supplement: Supplementary file 1 — Supplementary material 1 (PDF 1105 kb) [file 13280_2013_408_MOESM1_ESM.pdf]

Electronic Supplementary Material

Inferences about Radionuclide Mobility in Soils Based on the Solid/Liquid Partition Coefficients and Soil Properties

Gustav Sohlenius, Peter Saetre, Sara Nordén, Sara Grolander, Steve Sheppard

BOX S1: REGOLITH IN THE FORSMARK AREA TODAY AND IN THE FUTURE

In the Forsmark area (Fig. S1), as in other parts of Sweden, most regolith (unconsolidated deposit) was formed during or after the final phase of the latest glaciation. After the deglaciation, ~8800 BC (Fredén 2002), the area was covered by water of the Baltic Basin, due to the isostatic depression caused by the ice sheet (Påsse and Andersson 2005; Söderbäck 2008).

Since then the water has receded as an effect of the isostatic land uplift and, during the last two thousand years, there has been a significant increase in land area in the Forsmark region. The land uplift is still ongoing and since the area is flat the increase of new land is relatively fast even though the land uplift is only 6 mm/year. Lakes and wetlands are continuously formed in the uplifted areas. The lakes are slowly filled up with sediment and peat and are likely to develop into fens, which in the future may be cultivated (Lindborg et al. 2013).

The regolith types used for agriculture today are most commonly characterized by high clay content, which is the case with the clayey till, glacial clay, and clay gyttja studied here. The clayey till was deposited directly by the ice and is, in the Forsmark area, characterized by a high content of $CaCO_3$ (around 20%). In parts of the area, the till also has high clay content and a low content of boulders and stones, making it suitable for agriculture.

After deglaciation, glacial clay was deposited at the floor of the Baltic. This glacial clay was deposited shortly after deglaciation and is characterized by a high content of $CaCO_3$. Later postglacial clays started to accumulate and are continuously deposited at the deepest bottoms and in sheltered bays. These clays often contain a significant amount of organic material and are then referred to as clay gyttja.

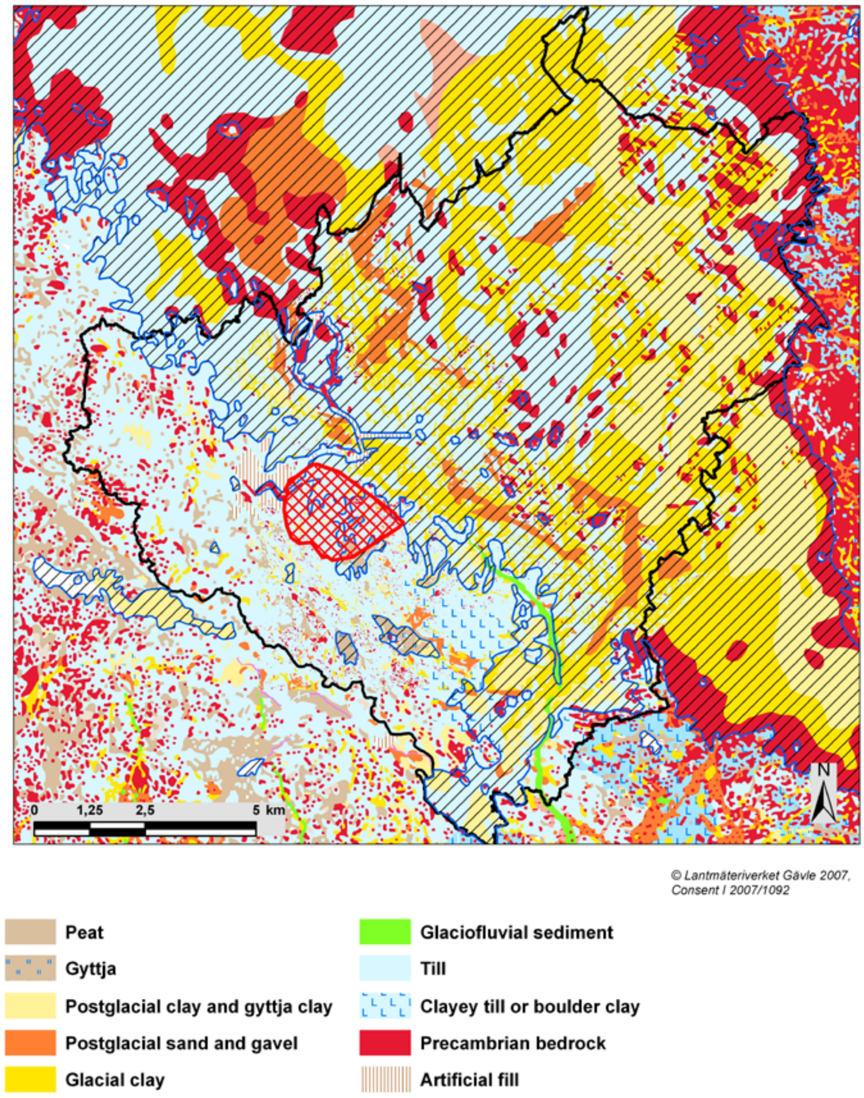

Fig. S1 The present distribution of regolith in the Forsmark area (from Hedenström and Sohlenius 2008). The red hatching represents the location of the planned geological repository for radioactive wastes. The black line represents the extent of the area included in the landscape model (Lindborg et al. 2013) predicting the future distribution of land use in the Forsmark area. The areas with diagonal lines are presently covered by water. A large proportion of the present sea floor is covered with clays. In the future, these areas will be uplifted and may then be used as arable land.

Peat is, in some areas, used as arable land. This regolith type is almost entirely built up of organic material and is formed in wetlands which may be preceded by a lake stage.

The proportion of areas with clay will increase as the large-scale valley in 'Öregrundsgrepen' is uplifted (Fig. S1). In the present terrestrial area, the proportion of peat soils will increase significantly as the shallow lakes are gradually filled with organic sediments and the low-lying wetlands are covered by a layer of peat. Thus, the proportion of land suitable for agriculture will probably increase in the future as the areas with peat and clay increase (Lindborg et al. 2013).

Compared with most other parts of Sweden, regolith in the Forsmark area has only been subjected to soil-forming processes for a relatively short period and most of the soils are therefore immature and lack distinct soil horizons (Lundin et al. 2004). During the next few thousands of years, the present soils will successively develop into more mature soil types.

SOIL SAMPLING ILLUSTRATED

Samples were taken from each regolith type, at two different depths at each position.

At the sites used as arable land, samples were taken in spade dug pits whereas a peat corer was used in the wetlands – see Figure S2 (page 4 below).

APPENDIX 1: STATISTICAL TESTS

Table S1 (pages 5-7 below) presents statistical tests of the relationship between K_d values, regolith type, sampling depth, and soil properties for 69 elements in agricultural soils and natural wetland peat in the Forsmark region.

For details of the analyses, see the subsection 'Statistical analysis' in the 'Methods and materials' section of the main (printed) document.

REFERENCES

Fredén, C. (ed.) 2002. *Berg och jord.* Third edition. Stockholm: Sveriges nationalatlas. 208 pp. (In Swedish).

Hedenström, A., and G. Sohlenius. 2008. Description of the regolith at Forsmark. Site descriptive modelling SDM-Site Forsmark. Svensk Kärnbränslehantering AB, SKB R-08-04, Stockholm, Sweden, Report, 221 pp.

Lundin, L. E. Lode, J. Stendahl, P.A. Melkerud, L. Björkvald, and A. Thorstensson. 2004. Soils and site types in the Forsmark area. Svensk Kärnbränslehantering AB, SKB R-04-08, Stockholm, Sweden, Report, 102 pp.

Påsse, T., and L. Andersson. 2005. Shore –level displacement in Fennoscandia calculated from empirical data. *Geologiska Föreningen i Stockholm Förhandlingar* 127: 253-268.

Söderbäck, B. 2008. Geological evolution, palaeoclimatology and historic development of the Forsmark and Laxemar/Simpevarp areas. Site descriptive modelling SDM-Site. Svensk Kärnbränslehantering AB, SKB R-08-19, Stockholm, Sweden, Report, 228 pp.

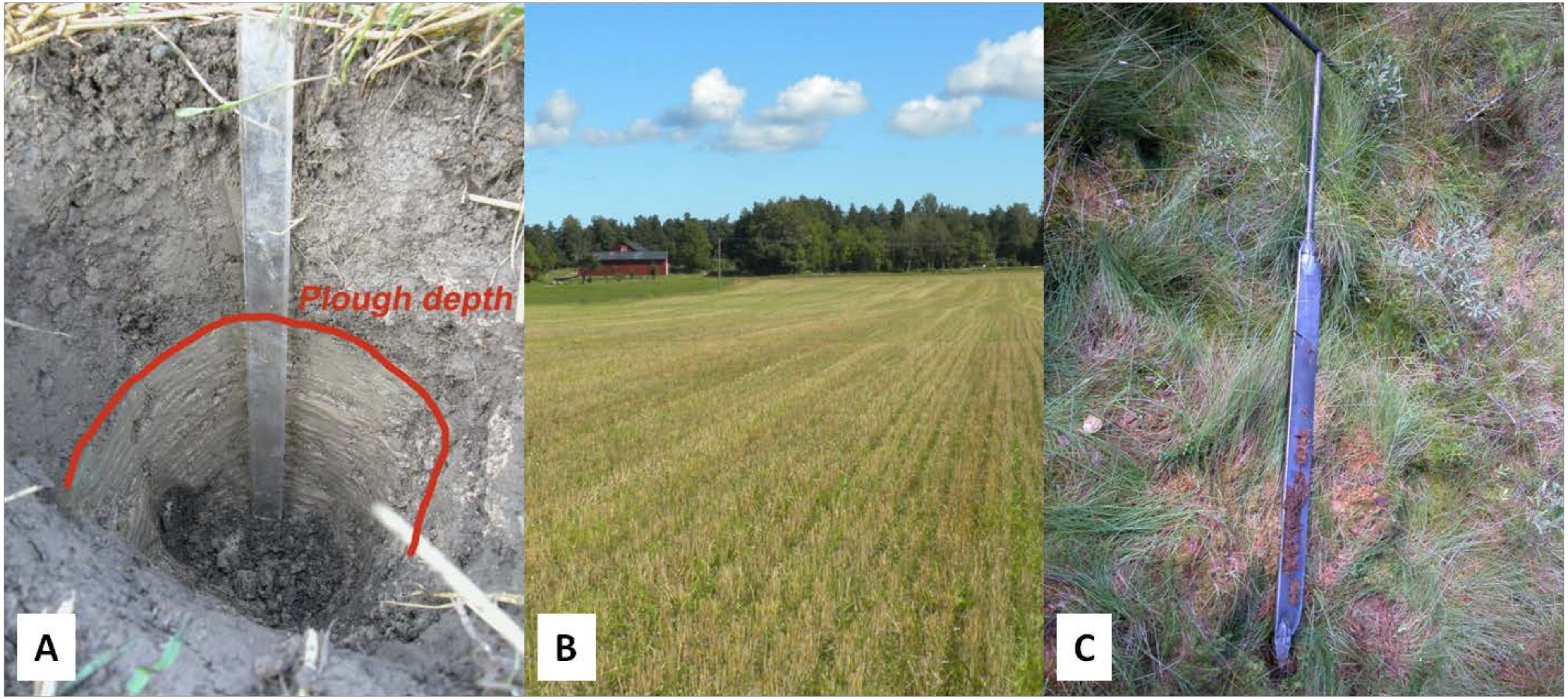

Fig. S2

a One of the pits from which samples for analysis were taken. The red line represents the depth affected by plowing. One sample was taken from within the plough depth and one from below that depth. The deposit shown here is glacial clay, which below the plow depth is characterized by light and dark layers representing the annual sedimentation during the latest deglaciation

b One of the sites where clayey till was sampled

c Peat from one of the studied wetlands, which was sampled with a Russian peat corer

Table S1 Statistical tests of the relationship between K_d values, regolith type, sampling depth, and soil properties for 69 elements in agricultural soils and natural wetland peat in the Forsmark region. **Left part**: F-ratios from split-plot analysis of variance of the effect of QD type, depth, and their interaction, on K_d-values. **Right part**: t-values from stepwise multiple regression of K_d-values as a function of soil chemical properties (right). Statistical significance indicated by asterisks (* p>0.01, ** p<0.001, *** p<0.0001). Elements have been grouped into clusters with respect to similarity in K_d profile

			**ANOVA (F-ratios)**			**Stepwise regression (t-values)**								
**Cluster**	**Element**	**N**	**QD type**	**Depth**	**Depth×QD**	**pH_{H2O}**	**Org C (%)**	**Clay (%)**	**Al cmol/kg**	**Na cmol/kg**	**Acidity cmol/kg**	**K cmol/kg**	**CEC cmol/kg**	**r^2_{model}**
**1.**	**Ga**	50	20.8 ***	9.7 *	4.6 *	4.4 **	-1.8 **	2.7 **	2.6 **	.	.	.	.	0.76
	**Ti**	50	17.8 ***	18.3 **	6.1 *	4.3 **	-3.4 **	2.5 **	.	.	.	.	.	0.58
	**Nb**	50	15.3 ***	27.6 ***	6.6 *	8.1 **	.	2.8 **	4.5 **	.	2.1 **	.	.	0.55
	**V**	50	12.4 ***	40.2 ***	7.9 **	4.5 **	0.6 **	5.5 **	2.9 **	.	.	.	.	0.56
	**As**	50	28.5 ***	6.5	8.1 **	.	-10.1 **	3.7 **	.	.	.	.	.	0.71
	**Cl**	48	9.8 **	0.1	1.6	.	6.4 **	.	2.4 **	.	.	-2.5 **	.	0.65
	**W**	49	0.5	5.4	9.1 **	.	.	.	.	.	.	2.1 **	.	0.17
	**Si**	50	4.6 *	0.1	1.8	. **	-4.0 **	.	.	.	.	.	.	0.25
	**Sn**	50	1.5	0.0	1.0	.	.	.	.	.	-3.2 **	3.2 **	.	0.28
	**Ru**	25	0.4	0.3	1.0	. **	.	.	.	.	.	.	.	0.00
**2.**	**Cs**	50	66.6 ***	0.9	1.0	2.9 **	-13.2 **	.	.	-4.0 **	-2.1 **	.	.	0.91
	**Rb**	50	44.2 ***	1.5	0.7	. **	-13.8 **	2.7 **	.	-7.0 **	.	.	2.6	0.91
	**Li**	50	25.9 ***	0.5	1.4	. **	-9.3 **	.	.	-2.8 **	.	.	.	0.72
	**Tl**	50	14.8 ***	2.6	4.3	5.3 **	-2.5 **	.	2.1 **	.	.	4.8 **	.	0.86
	**Mg**	50	10.6 ***	24.7 ***	2.9	. **	-9.4 **	.	.	.	.	.	.	0.65
	**Na**	50	4.9 *	10.9 *	0.9	. **	-4.7 **	. **	.	-2.3 **	.	.	.	0.57
**3.**	**Cd**	34	14.3 ***	0.1	3.5	.	3.1 **	.	-6.4 **	.	.	.	.	0.85
	**B**	50	6.8 *	0.1	6.6 *	5.1 **	5.8 **	.	.	.	-3.2 **	4.5 **	1.0 **	0.85
	**Ca**	50	8.8 **	26.3 ***	15.2 ***	.	.	2.6 **	-6.0 **	.	.	.	.	0.68
	**Sr**	50	4.5 *	44.4 ***	19.4 ***	2.1 **	.	4.4 **	-3.3 **	-2.3 **	3.8 **	.	.	0.57
	**Cu**	50	21.0 ***	6.4	4.4	.	.	3.2 **	-2.2 **	.	-2.1 **	.	5.8 **	0.63
	**Au**	35	1.6	0.0	0.8	.	.	2.3 **		-4.9 **	2.1 **	.	4.1 **	0.59

Table cont'd next page

Table S1 cont'd

			ANOVA (F-ratios)			Stepwise Regression (t-values)								
**Cluster**	**Element**	**N**	**QD type**	**Depth**	**Depth×QD**	**pH_{H2O}**	**Org C (%)**	**Clay (%)**	**Al cmol/kg**	**Na cmol/kg**	**Acidity cmol/kg**	**K cmol/kg**	**CEC cmol/kg**	**r^2_{model}**
**4.**	**Al**	50	17.9 ***	10.0 *	11.3 ***	12.7 **	.		2.9 **	.	.	2.6	.	0.85
	**Fe**	50	16.3 ***	22.2 **	9.6 **	9.3 **	.	2.7 **	2.1 **	.	.	.	.	0.68
	**Sc**	50	39.9 ***	5.1	7.8 **	9.7 **	-3.3 **	3.6 **	.	-3.4 **	.	.	.	0.85
	**Th**	50	23.9 ***	12.7 *	11.4 ***	9.3 **	.	**	3.8 **	.	.	.	.	0.66
	**Pb**	50	11.3 ***	5.3	10.6 ***	8.3 **	.		.	.	.	.	.	0.59
	**Cr**	50	21.8 ***	6.4	1.5	5.5 **	-3.6 **		.	.	.	.	.	0.64
	**Y**	50	17.7 ***	8.2 *	15.7 ***	12.6 **	4.9 **	3.1 **	.	.	.	.	.	0.78
	**Be**	50	26.2 ***	1.0	5.4 *	10.0 **	-2.6 **	5.0 **	.	-5.9 **	.	.	.	0.86
	**Zn**	50	14.0 ***	0.3	7.8 **	10.5 **	.	5.3 **	-3.4 **	.	.	7.2 **	.	0.89
**5.**	**S**	50	22.4 ***	2.8	2.1	. **	3.9 **		.	.	.	2.4	1.2 **	0.73
	**K**	50	15.7 ***	6.2	2.8	. **	-12.3 **	7.1 **	.	.	.	-5.7 **	.	0.81
	**Re**	49	9.4 **	0.4	5.3 *	. **	6.9 **		.	.	.	.	.	0.50
	**Hg**	50	7.4 **	0.7	1.9	. **	4.9 **	**	.	.	.	.	.	0.33
	**Ag**	50	9.8 **	15.9 **	3.1	.	4.1 **		.	.	.	.	.	0.44
	**Os**	32	1.0	0.0	0.7	. **	.		.	.	.	.	.	0.00
**6.**	**U**	50	27.8 ***	0.4	0.6	.	13.1 **	**	4.5 **	.	.	.	.	0.83
	**Mo**	50	31.3 ***	5.0	4.0	-7.5 **	.	**	.	4.9 **	.	.	.	0.73
	**Br**	50	27.7 ***	8.7 *	9.0 **	-3.8 **	2.8 **		.	.	.	.	3.1 **	0.73
	**Sb**	50	15.9 ***	7.9	5.1 *	. **	.	6.6 **	.	.	.	.	6.8 **	0.68
	**I**	50	2.4	18.3 **	4.6 *	. **	2.2 **		.	.	.	.	.	0.09
	**Se**	30	1.1	7.2	2.2	. **	.	5.2 **	.	-3.5 **	.	.	.	0.62

Table cont'd next page

Table S1 cont'd

			ANOVA (F-ratios)			Stepwise Regression (t-values)								
**Cluster**	**Element**	**N**	**QD type**	**Depth**	**Depth×QD**	**pH$_{H2O}$**	**Org C (%)**	**Clay (%)**	**Al cmol/kg**	**Na cmol/kg**	**Acidity cmol/kg**	**K cmol/kg**	**CEC cmol/kg**	**r$^2_{model}$**
**7.**	**Hf**	50	8.5 **	54.2 ***	17.7 ***	7.8 **	.	5.0 **	.	.	.	.	.	0.52
	**Zr**	50	8.4 **	51.4 ***	18.7 ***	7.2 **	.	5.2 **	.	.	.	.	.	0.47
	**Bi**	50	7.9 **	12.6 *	8.1 **	5.7 **	.	2.7 **	.	.	.	.	.	0.42
	**Ta**	48	1.8	11.9 *	4.9 *	2.3 **	.	.	.	.	.	.	.	0.23
	**Te**	50	3.0	7.8	3.9	4.9 **	.	5.0 **	.	.	.	.	.	0.50
	**P**	50	1.2	0.4	3.1	2.9 **	.	2.5 **	.	.	.	.	.	0.21
	**Ge**	50	1.8	1.2	0.7	. **	.	.	.	.	.	.	.	0.00
**8.**	**Mn**	50	34.9 ***	38.0 ***	13.0 ***	10.7 **	-2.9 **	2.7 **	.	-3.2 **	4.3 **	.	.	0.84
	**Co**	50	50.0 ***	21.1 **	9.7 **	15.5 **	.	5.9 **	.	-2.6 **	5.0 **	.	.	0.87
	**Ni**	50	20.1 ***	29.4 ***	13.6 ***	7.1 **	.	5.6 **	-2.8 **	.	2.4 **	.	.	0.78
	**Ra^{226}**	48	4.4	5.7	6.7 *	2.4 **	-0.7 **	.	1.0	.	3.3 **	.	.	0.58
	**Ba**	50	2.3	67.3 ***	14.1 ***	3.8 **	-2.5 **	5.5 **	.	-2.8 **	3.6 **	.	.	0.45
**Lanth.**	**Dy**	50	16.8 ***	8.9 *	13.3 ***	11.8 **	4.1 **	3.2 **	.	.	.	.	.	0.77
	**Ho**	50	17.7 ***	8.7 *	12.8 ***	11.6 **	4.3 **	3.3 **	.	.	.	.	.	0.76
	**Tb**	50	15.5 ***	11.8 *	17.0 ***	11.9 **	4.1 **	3.2 **	.	.	.	.	.	0.79
	**Er**	50	17.6 ***	8.0	12.3 ***	11.8 **	4.2 **	3.2 **	.	.	.	.	.	0.76
	**Tm**	50	17.3 ***	7.5	10.3 **	12.1 **	4.3 **	3.5 **	.	.	.	.	.	0.77
	**Gd**	50	16.6 ***	10.7 *	14.7 ***	11.6 **	4.0 **	3.0 **	.	.	.	.	.	0.78
	**Nd**	50	17.8 ***	10.3 *	14.2 ***	12.0 **	3.8 **	2.7 **	.	.	.	.	.	0.79
	**Sm**	50	16.7 ***	9.4 *	13.8 ***	11.6 **	3.9 **	2.8 **	.	.	.	.	.	0.79
	**Yb**	50	17.4 ***	8.4 *	10.2 **	11.9 **	4.3 **	3.2 **	.	.	.	.	.	0.76
	**Pr**	50	17.5 ***	9.2 *	15.0 ***	12.0 **	3.8 **	2.5 **	.	.	.	.	.	0.79
	**Lu**	50	17.7 ***	7.2	9.8 **	11.7 **	4.4 **	3.3 **	.	.	.	.	.	0.76
	**La**	50	18.9 ***	7.5	16.6 ***	12.2 **	3.7 **	2.5 **	.	.	.	.	.	0.79
	**Ce**	50	22.0 ***	5.8	14.7 ***	11.2 **	2.5 **	.	.	.	.	.	.	0.77
	**Eu**	29	7.2 *	2.4	5.0	7.0 **	.	.	.	.	.	.	.	0.64
